# Oncolytic Reovirus Infection Is Facilitated by the Autophagic Machinery

**DOI:** 10.3390/v9100266

**Published:** 2017-09-21

**Authors:** Vera Kemp, Iris J. C. Dautzenberg, Ronald W. Limpens, Diana J. M. van den Wollenberg, Rob C. Hoeben

**Affiliations:** Department of Molecular Cell Biology, Leiden University Medical Center, P.O. Box 9600, 2300 RC Leiden, The Netherlands; v.kemp@lumc.nl (V.K.); i.j.c.dautzenberg@lumc.nl (I.J.C.D.); r.w.a.l.limpens@lumc.nl (R.W.L.); d.j.m.van_den_wollenberg@lumc.nl (D.J.M.v.d.W.)

**Keywords:** mammalian orthoreovirus, replication, autophagy machinery, knockout, oncolysis

## Abstract

Mammalian reovirus is a double-stranded RNA virus that selectively infects and lyses transformed cells, making it an attractive oncolytic agent. Despite clinical evidence for anti-tumor activity, its efficacy as a stand-alone therapy remains to be improved. The success of future trials can be greatly influenced by the identification and the regulation of the cellular pathways that are important for reovirus replication and oncolysis. Here, we demonstrate that reovirus induces autophagy in several cell lines, evident from the formation of Atg5-Atg12 complexes, microtubule-associated protein 1 light chain 3 (LC3) lipidation, p62 degradation, the appearance of acidic vesicular organelles, and LC3 puncta. Furthermore, in electron microscopic images of reovirus-infected cells, autophagosomes were observed without evident association with viral factories. Using UV-inactivated reovirus, we demonstrate that a productive reovirus infection facilitates the induction of autophagy. Importantly, knock-out cell lines for specific autophagy-related genes revealed that the expression of Atg3 and Atg5 but not Atg13 facilitates reovirus replication. These findings highlight a central and Atg13-independent role for the autophagy machinery in facilitating reovirus infection and contribute to a better understanding of reovirus-host interactions.

## 1. Introduction

Mammalian orthoreovirus, henceforth referred to as reovirus, is broadly studied as an anti-cancer agent both as a monotherapy and in combination with existing therapies [1]. It has the natural preference to replicate in and lyze tumor cells, while an antiviral response in normal cells hinders virus replication and cytolysis. The name “reovirus” is an acronym for Respiratory and Enteric Orphan virus, as it infects the respiratory and enteric tract and has not been associated with serious disease in humans. Reovirus is one of the first viruses for which a molecular mechanism has been suggested to explain its tumor cell preference [2]. This mechanism contributed to its clinical evaluation as viral oncolytic agent. To date a variety of clinical trials have been completed in several cancer types, but while the virus administration has been found safe, its efficacy in stand-alone treatments remains to be improved. A better understanding of what intracellular factors and pathways are important to reovirus replication and oncolysis would facilitate the improved design of clinical studies.

Various viruses have been shown to induce a host-cell adaptive response called macroautophagy, hereafter referred to as autophagy [3]. During autophagy, the cytoplasmic cellular contents are sequestered within double-membraned vesicles termed autophagosomes, which ultimately fuse to endosomes or lysosomes to form amphisomes or autolysosomes, respectively. This process facilitates the degradation of the cellular contents, even whole organelles, upon which the degradation products can be shuttled back into the cytosol for recycling. This highly conserved homeostatic process allows the cell to survive stressful conditions such as a nutrient-poor environment. Autophagy can also be exploited to combat viral infections. For instance, it can promote the intracytoplasmic degradation of viruses such as Sindbis virus and HIV-1 [4,5]. Alternatively, it can activate an antiviral immune response through the delivery of viral genomic components to endosomal Toll-Like Receptors (TLRs) or through the facilitation of viral antigen presentation on major histocompatibility complex (MHC) molecules [3,6]. On the other hand, it has been demonstrated that viruses have evolved ways to either suppress or induce the autophagy machinery to facilitate their replication and/or survival [3]. For example, autophagy facilitates cancer cell death induction by human adenovirus type 5, presumably through the triggering of caspase activity [7]. Autophagy has also been shown to facilitate the infection of several dsRNA virus family members. Rotavirus induces microtubule-associated protein 1 light chain 3 (LC3) lipidation and inhibition of this process decreases virus replication [8]. Interestingly, Rotavirus does not induce the formation of autophagosomes. Furthermore, the non-structural avian reovirus protein p17 triggers autophagy, which enhances virus replication [9]. For Bluetongue virus, a similar correlation has been found, as inhibition of autophagy decreases viral protein production and virus titer, and the stimulation of autophagy conversely resulted in increased viral protein synthesis and virus yields [10]. It has been suggested that mammalian reovirus induces autophagy as well, though the precise function during reovirus infection remained unclear [11,12].

In the present study, we show that reovirus induces the full autophagic flux in immortalized mouse embryonic fibroblasts. The presence of a distinct set but not all of the autophagy-related proteins seems to facilitate reovirus replication. Importantly, autophagic features could also be observed in human glioblastoma cell lines. Moreover, a productive reovirus infection facilitates the induction of autophagy.

## 2. Materials and Methods

### 2.1. Reagents and Buffers

Rapamycin (Rapa) and Bafilomycin A1 (BafA1) were purchased from Sigma-Aldrich (St. Louis, MO, USA). Stock solutions were stored at −20 °C. Rapa was reconstituted in pure ethanol at a concentration of 1 mM, and BafA1 in pure ethanol at a concentration of 50 µM. Acridine orange (Sigma-Aldrich) was reconstituted in milli-Q at a concentration of 2 mM.

RIPA lysis buffer contains 50 mM Tris·HCl pH 7.5, 150 mM sodium chloride, 0.1% sodium dodecyl sulphate, 0.5% sodium deoxycholate, and 1% NP40. Giordano lysis buffer contains 50 mM Tris·HCl pH 7.4, 250 mM sodium chloride, 0.1% Triton X-100, and 5 mM EDTA. Lysis buffers were supplemented with protease inhibitors (Complete mini tablets, Roche Diagnostics, Almere, The Netherlands). Western sample buffer has the following concentrations: 50 mM Tris·HCl pH 6.8, 10% glycerol, 2.5% β-mercaptoethanol, 2% SDS, and 0.025% bromophenol blue.

### 2.2. Cell Lines

Human embryonic kidney 293T [13], embryonic retinoblast-derived 911 [14], glioblastoma U87-MG (ATCC), and U251-MG (ATCC) cell lines were cultured at 37 °C in high-glucose Dulbecco’s Modified Eagle Medium (DMEM) (Invitrogen, Breda, The Netherlands), supplemented with penicillin, streptomycin (pen-strep), and 8% fetal bovine serum (FBS) (Invitrogen, Breda, The Netherlands). Immortalized Atg3−/− and the control wildtype Atg3wt mouse embryonic fibroblasts (MEFs) were kindly provided by Masaaki Komatsu, the Tokyo Metropolitan Institute Medical Science [15]; Atg5−/−, and the wildtype Atg5wt MEFs by Noboru Mizushima, the University of Tokyo [16]; Atg13−/− with the control Atg13wt MEFs by Xiaodong Wang, Beijing National Institute of Biological Sciences [17]. Atg3 and Atg5 genes were reintroduced in the immortalized knock-out MEFs by lentiviral or retroviral transductions. Lentivirus and retrovirus was produced by standard production protocols. MEFs with a reintroduced Atg13 gene were kindly provided by Fulvio Reggiori (University Medical Center Groningen, Groningen, The Netherlands), and were previously described in literature [18]. All MEFs were cultured in DMEM plus pen-strep and 10% FBS. The Atg3wt, Atg3−/−, and Atg3+/+ MEFs were cultured at 32 °C, and all other MEFs at 37 °C. All cell lines were maintained in a 5% CO_2_ atmosphere.

### 2.3. Viruses

The wild-type type 3 Dearing (T3D) strain R124 was isolated from a reovirus T3D stock obtained from ATCC and propagated as previously described [19]. The jin-mutant reoviruses were isolated from U118MG cells upon infection with wildtype T3D [19]. Infectious virus titers were determined by plaque assays on 911 cells. All experiments were performed with CsCl-purified virus stocks.

For UV inactivation, R124 was reconstituted at a concentration of 5.95 × 10^9^ pfu/mL in MEM supplemented with pen-strep and 2% FBS. UV inactivation was achieved by exposure to shortwave UV light for 15 min. The absence of infectious virus was confirmed by plaque assay on 911 cells (≤10 pfu/mL).

### 2.4. Yield Quantification

To determine the replication of R124 and jin-3 in MEFs, cells were seeded in 24-well plates at a density of 1 × 10^5^ cells/well. Infections with R124 or jin-3 reoviruses were performed at the indicated multiplicity of infection (MOI), in DMEM containing 2% FBS and pen-strep. After the indicated time periods, when CPE became clearly visible, reoviruses were harvested from the cells and medium by three freeze-thaw cycles. Virus yields were quantified by plaque assays on 911 cells.

### 2.5. Western Blotting

For western blot analysis, the total protein concentrations were determined by a BCA assay kit (Thermo Scientific Pierce, Rockford, IL, USA). Equal amounts of protein (30 µg) were separated by gel electrophoresis on gels with the indicated percentage of polyacrylamide, and transferred onto Immobilon-P membranes (Millipore, Etten-Leur, The Netherlands). The proteins were visualized using standard protocols. Primary antibodies rabbit anti-Atg5 (ab108327), mouse anti-LC3B (ab129376), rabbit anti-Atg3 (EPR4801) (Abcam, Cambridge, UK), mouse anti-p62 (ab56416) (Abcam, Cambridge, UK) were purchased from Abcam, rabbit anti-LC3B (NB600-1384) from Novus Biologicals (Littleton, CO, USA), mouse anti-βactin from MP Biomedicals (Santa Ana, CA, USA) and rabbit anti-Atg13 from Sigma-Aldrich (St. Louis, MO, USA). Primary 4F2 mouse anti-reovirus σ3 was obtained from the Developmental Studies Hybridoma Bank developed under the auspices of the NICHD and maintained by the University of Iowa, Department of Biology (Iowa City, IA, USA) [20].

### 2.6. eGFP-LC3 Fluorescence Microscopy

The eGFP-LC3 fusion orf from plasmid Addgene #11546 was cloned downstream of the internal CMV promoter of plasmid pLV-CMV-IRES Puro expressing a puromycin-resistance cassette. Atg3wt MEFs were transduced with the lentivirus, and puromycin selection was performed. The resulting eGFP-LC3 expressing Atg3wt MEFs will be referred to as Atg3wt-G MEFs. To analyze the localization of LC3, Atg3wt-G MEFs were seeded on cover slips in 24-wells plates at 100,000 cells/well, and infected with R124 or jin-3 at an MOI of 10. As a positive control, cells were treated with Rapa at a concentration of 1 µM, and 50 nM BafA1 was supplemented 16 h before the end of the experiment. After 72 h, the cells were fixed in 4% paraformaldehyde (PFA), permeabilized with 0.5% Triton, blocked with 3% BSA, and stained with primary and secondary antibodies. The primary antibody used is the 10F6 mouse anti-µ1C antibody, obtained from the Developmental Studies Hybridoma Bank developed under the auspices of the NICHD and maintained by the University of Iowa, Department of Biology (Iowa City, IA, USA) [20]. A647-coupled anti-mouse antibody was used as a secondary antibody. After the antibody stainings, the coverslips were incubated in anti-fade reagent (ProLong Gold with DAPI, Cell Signaling Technology, Leiden, The Netherlands) on a glass microscopic slide, and imaged the next day using a Leica DM6B fluorescence microscope (Wetzlar, Germany) and home-made ColourProc software (LUMC, Leiden, The Netherlands).

### 2.7. Acridine Orange Staining

To examine the presence of acidic vesicular organelles, U87-MG cells were grown on cover slips in 24-wells plates at 100,000 cells/well and exposed to R124 at an MOI of 10. Alternatively, cells were treated with 400 nM Rapa. After 48 h, acridine orange was added (15 min, 37 °C). The cover slips were transferred to glass microscopic slides and analyzed by fluorescence microscopy using a Leica DMRA fluorescence microscope and ColourProc software.

### 2.8. Electron Microscopy

For electron microscopy analysis, U87-MG cells were seeded in 10 cm dishes at 3 × 10^6^ cells/dish and exposed to R124 at an MOI of 10 or 400 nM Rapa. After 48 h, cells were fixed in 1.5% glutaraldehyde in 0.1 M Cacodylate buffer (1 h, room temperature), post-fixed in 1% OsO_4_ in 0.1 M Cacodylate buffer (1 h, 4 °C), dehydrated in a graded ethanol series, and embedded in epon. Ultrathin cell sections (100 nm) were post-stained with uranyl acetate and lead citrate. Analyses were performed using a Tecnai 12 electron microscope at 120 kV equipped with a 4K Eagle CCD Camera (FEI Company, Eindhoven, The Netherlands).

## 3. Results

### 3.1. Reovirus Induces LC3 Puncta in MEFs

**Figure 1:**
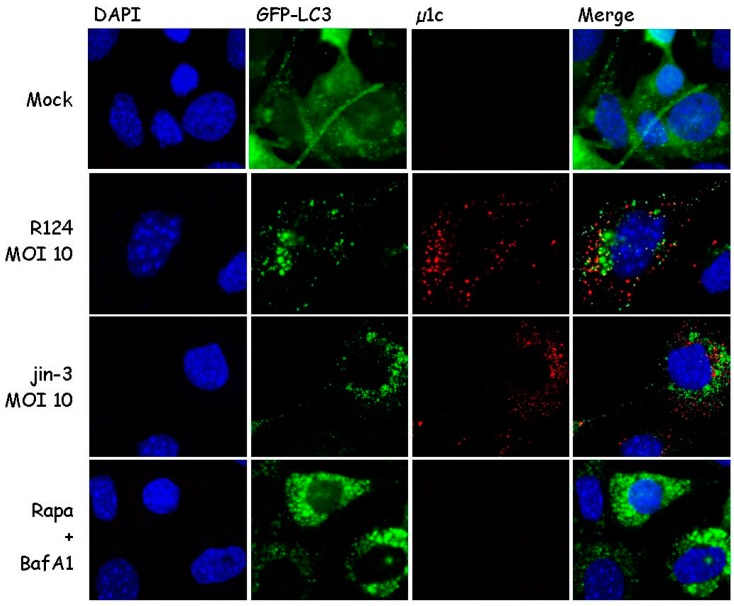
Reovirus infection triggers microtubule-associated protein 1 light chain 3 (LC3) puncta in mouse embryonic fibroblasts (MEFs). MEFs were infected with R124 or jin-3 at a multiplicity of infection (MOI) of 10. Alternatively, cells were treated with 1 µM Rapamycin (Rapa), and after 32 h, 50 nM BafA1 was supplemented to the Rapa-treated cells. Cells were fixed after 48 h treatment and stained for reovirus µ1C protein in red. Cell nuclei are visible in blue (DAPI), and GFP-LC3 in green.

During autophagy, microtubule-associated protein 1 light chain 3 (LC3) is recruited to autophagosomal membranes [21]. To determine whether reovirus induces autophagy, the localization of LC3 was examined. To this end, mouse embryonic fibroblasts (MEFs) were stably transduced with the GFP-LC3 lentivirus and infected with R124 or jin-3 reovirus. R124 is our wildtype type 3 Dearing (T3D) strain, and jin-3 was obtained through serial passaging of the T3D reovirus on glioblastoma cells that lack the expression of the reovirus receptor junction adhesion molecule A (JAM-A) [19]. As a positive control, cells were treated with Rapamycin (Rapa) and Bafilomycin A1 (BafA1). Rapa is an mTOR inhibitor and a known autophagy inducer [21]. BafA1 blocks the acidification of lysosomes or the fusion between autophagosomes and lysosomes, and thereby arrests the autophagic flux through blocking the degradation of autophagosomes and their content. If autophagy is induced, the localization of the GFP-LC3 fusion protein shifts from a diffuse cytoplasmic appearance to a punctuate pattern. Immunofluorescent images revealed the induction of clear LC3 puncta upon reovirus infection and upon treatment with Rapa and BafA1 (Figure 1). Notably, LC3 puncta appeared to be present in cells that showed reovirus protein expression as well, as shown by a co-staining for reovirus protein µ1C. These findings suggest that reovirus induces autophagy.

### 3.2. Reovirus Induces Atg5-Atg12 Conjugation, p62 Degradation, and LC3 Lipidation in MEFs

**Figure 2:**
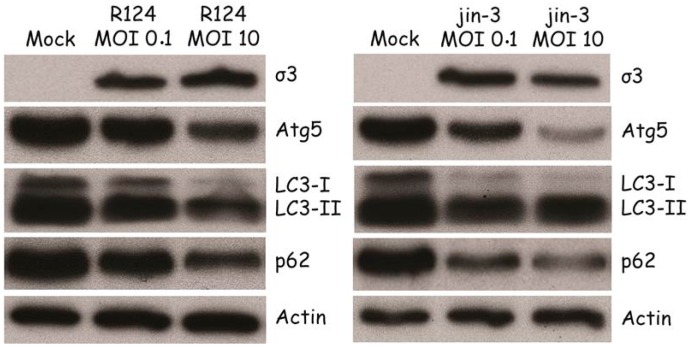
Reovirus infection induces Atg5-Atg12 conjugation, LC3-I to LC3-II conversion, and p62 degradation in MEFs. MEFs were infected with R124 or jin-3 at an MOI of 0.1 or 10. Protein lysates were prepared in Giordano lysis buffer 48 h post infection (hpi) and analyzed for the amounts of σ3, Atg5, LC3, p62, and Actin by western analysis.

Autophagy is characterized by the conjugation of Atg5 to Atg12 [21], resulting in a loss of free Atg5. Moreover, LC3-I is lipidated to form LC3-II. To examine whether reovirus induces these autophagic features, MEFs were infected with R124 or jin-3 and analyzed for the expression of free Atg5 and LC3. Reovirus infection clearly decreased the levels of free Atg5 and increased the conversion of LC3-I to LC3-II (Figure 2), most evident from the reduction in LC3 levels. These findings support the hypothesis that reovirus induces autophagy in MEFs. The jin strain seems to be a more potent inducer of autophagy than R124, probably caused by its ability to enter cells through a JAM-A independent mechanism. MEFs express modest levels of JAM-A on their cell surface hampering infection by R124 [22].

During the later steps in the autophagic process, p62 will be degraded inside the autophagolysosomes [21]. To establish whether the complete autophagic flux is induced by reovirus, p62 expression was determined. Upon infection with R124 and jin-3, a clear decrease in p62 levels could be detected, indicating that the full flux of autophagy was induced.

### 3.3. Atg3 and Atg5 but Not Atg13 Expression Facilitate Reovirus Replication

**Figure 3:**
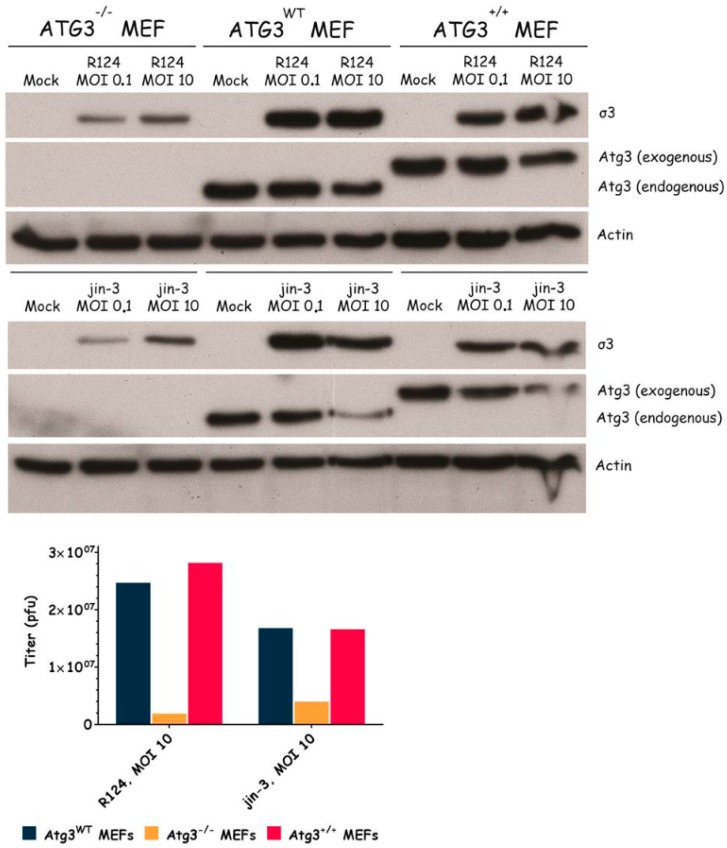
Reovirus replication in MEFs is facilitated by the expression of Atg3. Atg3wt, Atg3−/−, and Atg3+/+ MEFs were infected with R124 or jin-3 at an MOI of 0.1 or 10. After 48 h, protein lysates were prepared in Giordano lysis buffer and analyzed for the expression of σ3, Atg3, and Actin. In parallel, cells were collected in the supernatant, lyzed by three freeze-thaw cycles, and the total amount of infectious virus particles in the 24-wells plate well was determined by plaque assays.

**Figure 4:**
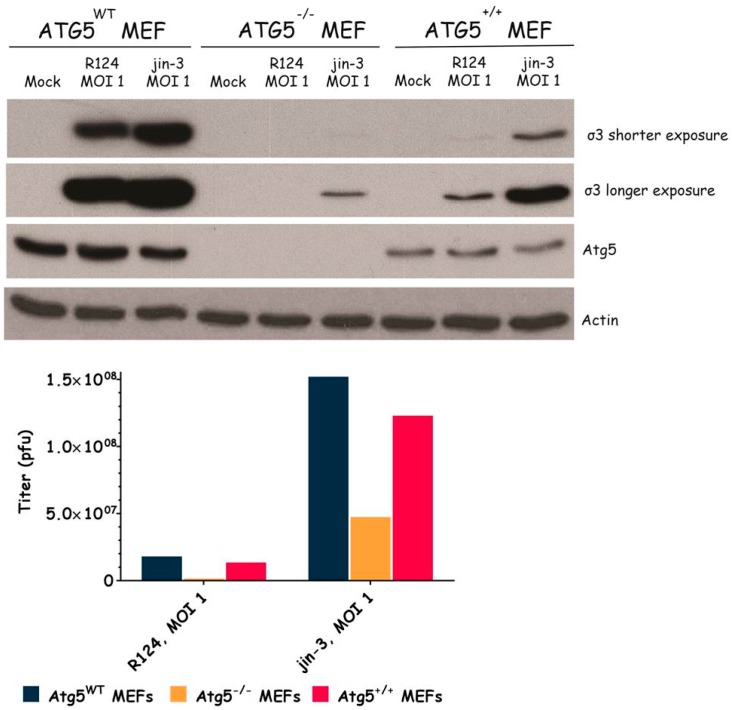
Reovirus replication is facilitated by the expression of Atg5. Atg5wt, Atg5−/−, and Atg5+/+ MEFs were infected with R124 or jin-3 at an MOI of 1. After 72 h, protein lysates were prepared in Giordano lysis buffer and analyzed for the expression of σ3, Atg5, and Actin. Alternatively, cells were collected in the supernatant, lyzed by three freeze-thaw cycles, and the total amount of infectious virus particles in the 24-wells plate well was determined by plaque assays.

**Figure 5:**
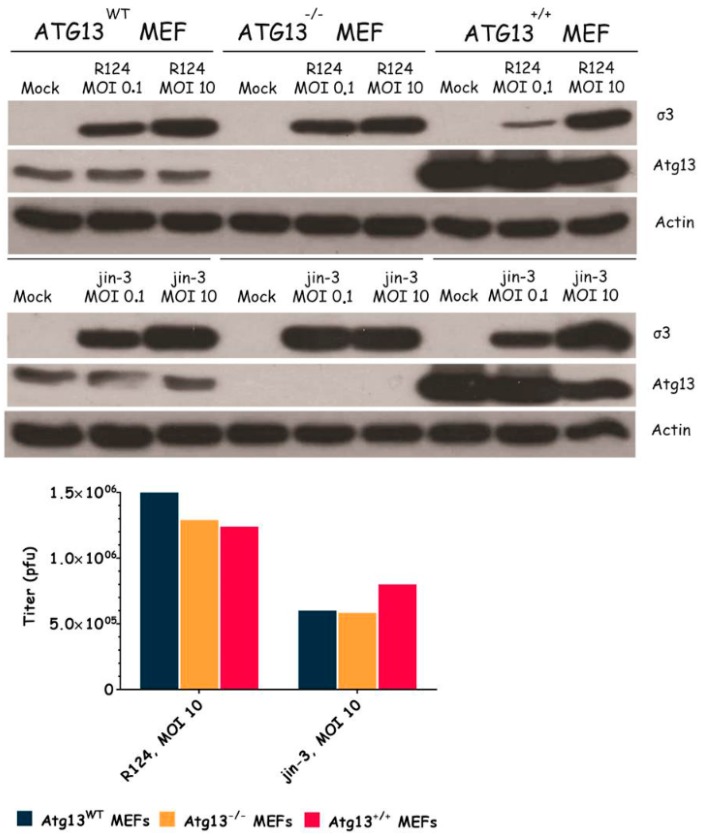
Reovirus replication is not influenced by Atg13 expression. Atg13wt, Atg13−/−, and Atg13+/+ MEFs were infected with R124 or jin-3 at an MOI of 0.1 or 10. After 48 h, protein lysates were prepared in Giordano lysis buffer and analyzed for the expression of σ3, Atg13, and Actin. In parallel, cells were collected in the supernatant, lyzed by three freeze-thaw cycles, and the total amount of infectious virus particles in the 24-wells plate well was determined by plaque assays.

To examine the importance of the autophagy machinery for reovirus infection, we used SV40 transformed MEF cell lines with a knock-out for a specific autophagy-related gene: Atg3, Atg5, or Atg13. Western blot results confirmed the absence of Atg3, Atg5, and Atg13 protein expression in the corresponding knock-out cell line (Figure 3, Figure 4 and Figure 5). We infected the MEFs with R124 or jin-3 and examined the cell viability, reovirus σ3 protein expression, and virus titer in the cells. For the different knock-outs, minor effects were seen on reovirus-induced cytolysis [23]. However, σ3 protein expression and virus titers were markedly decreased in MEFs lacking Atg3 or Atg5 compared to the corresponding wild-type and rescued cells (Figure 3 and Figure 4). A knock-out for Atg13 affected neither the reovirus σ3 expression nor the titer (Figure 5). Altogether, these results indicate that the expression of Atg3 and Atg5 but not Atg13 facilitates the replication of reovirus in SV40 transformed MEFs.

### 3.4. Reovirus Induces Atg5-Atg12 Conjugation, LC3 Lipidation, and Acidic Vesicular Organelles in Glioblastoma Cell Lines

**Figure 6:**
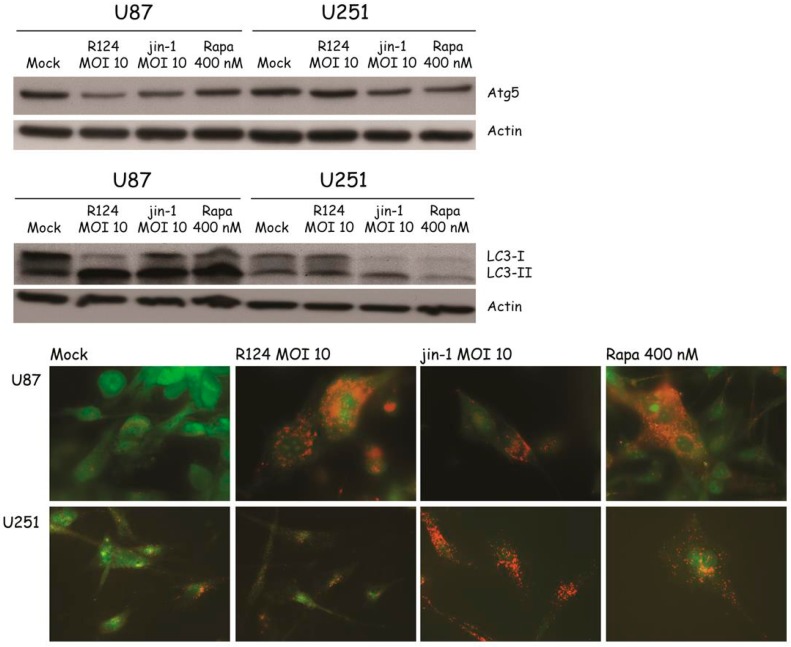
Reovirus infection induces Atg5-Atg12 conjugation, LC3-I to LC3-II conversion, and acidic vesicular organelles in U87-MG and U251-MG glioblastoma cells. U87-MG and U251-MG glioblastoma cells were infected with R124 or jin-1 at an MOI of 10, or treated with 400 nM Rapa. After 48 h (U87-MG) or 54 h (U251-MG), protein lysates were prepared in RIPA lysis buffer and analyzed for the expression of Atg5, LC3, and Actin. Alternatively, cells were incubated with acridine orange after 48 h (U87-MG) or 120 h (U251-MG), and fluorescence microscopy pictures were taken.

With the finding that reovirus induces several autophagic features in SV40 transformed MEFs, the question remains whether the same holds true for human cell lines. To study whether reovirus induces autophagy in human tumor cell lines, glioblastoma cell lines U251-MG and U87-MG were infected with R124 or jin-1, or stimulated with Rapa. Jin-1 was the first mutant reovirus we isolated from reovirus-infected JAM-A deficient glioblastoma cells [19]. At the indicated time points after treatment, protein lysates were made and analyzed for the expression of unconjugated Atg5 and LC3-I/LC3-II. Upon infection or Rapa treatment, Atg5 expression was decreased and the LC3-II/LC3-I ratio increased in U87-MG cells (Figure 6). U251-MG cells can be infected and killed efficiently by the jin reoviruses, but not R124 [24]. Corresponding to this, altered Atg5 and LC3-II expression was observed upon Rapa treatment and jin-1 but not R124 infection. Altogether, these data suggest that reovirus induces autophagy in human glioblastoma cell lines.

Autophagic vacuoles ultimately fuse to lysosomes, resulting in the formation of enlarged acidic vesicles in cells undergoing autophagy. To study the degree to which acidic vesicles are present in U87-MG and U251-MG cells upon reovirus infection, acridine orange was used. When added to cells, this dye diffuses throughout the cell, and once in an acidic environment, it becomes protonated and trapped. Upon excitation with UV light, the dye fluoresces green in neutral environments, and orange-to-red in acidic compartments. To study whether reovirus infection induces the formation of enlarged acidic vesicles, U87-MG and U251-MG cells were treated with R124, jin-1, or Rapa as before, incubated with acridine orange at the indicated time points and analyzed by fluorescence microscopy. Upon reovirus infection or Rapa treatment, enlarged orange-to-red vesicles were clearly visible in the U87-MG cells, indicating that reovirus induces the formation of autophagolysosomes. As for the Atg5 and LC3 expression, acidic vesicles were detected in U251-MG upon Rapa treatment and jin-1 but not R124 infection, as expected. Taken together, our data indicate that both R124 and jin-1 are able to induce autophagy in human glioblastoma cell lines.

### 3.5. Autophagosomes Can Be Observed in Reovirus-Infected U87-MG Cells

**Figure 7:**
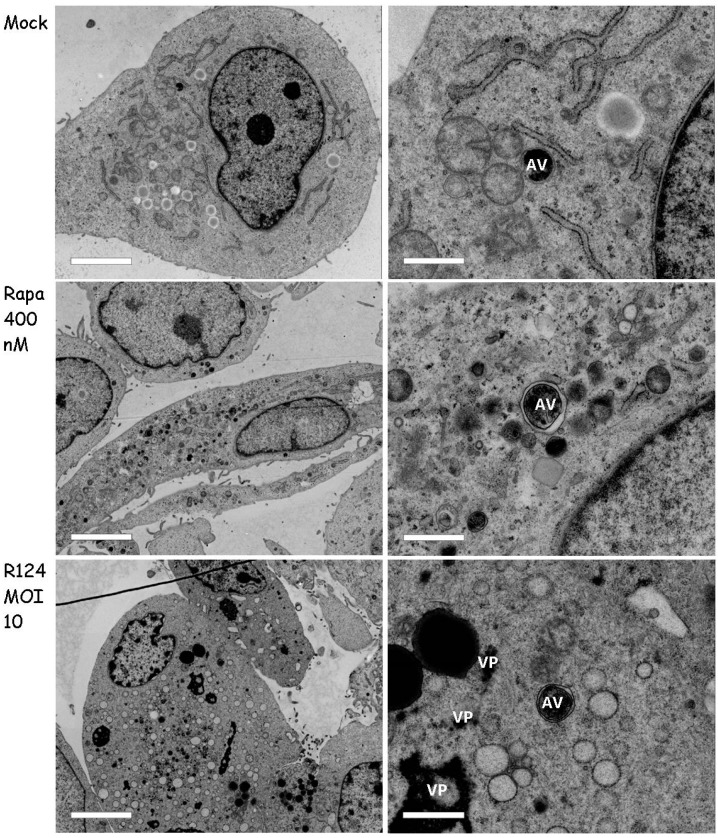
Autophagosomes can be observed in R124-infected U87-MG cells. U87-MG glioblastoma cells were left untreated (Mock) or were treated with 400 nM Rapa, or infected with R124 at an MOI of 10. After 48 h, cells were fixed, dehydrated, embedded, and ultrathin cell sections were analyzed by electron microscopy. Scale bars represent 4.6 µm (left panel), or 920 nm (right panel, 5× zoom of left panel cell). AV, autophagic vacuoles; VP, viral particles.

To further study the presence of double-membraned autophagosomes, we used electron microscopy. To this end, U87-MG cells were infected with R124 or treated with 400 nM Rapa as before, and analyzed by transmission electron microscopy. The results revealed the presence of viral replication factories upon R124 infection (Figure 7), indicative for a productive infection. Autophagosome-like structures could be observed in mock-treated U87-MG cells, and seemed to be enriched upon Rapa treatment. Importantly, such vesicles were present in reovirus-infected cells, without evident localization in close proximity of the viral factories. This suggests that the autophagosomes are not used to compartmentalize reovirus double-stranded RNA and reovirus assembly from the cytosol.

### 3.6. Reovirus Replication Facilitates the Induction of Acidic Vesicular Organelles, Atg5-Atg12 Conjugation, p62 Degradation and LC3 Lipidation in U87-MG Cells

**Figure 8:**
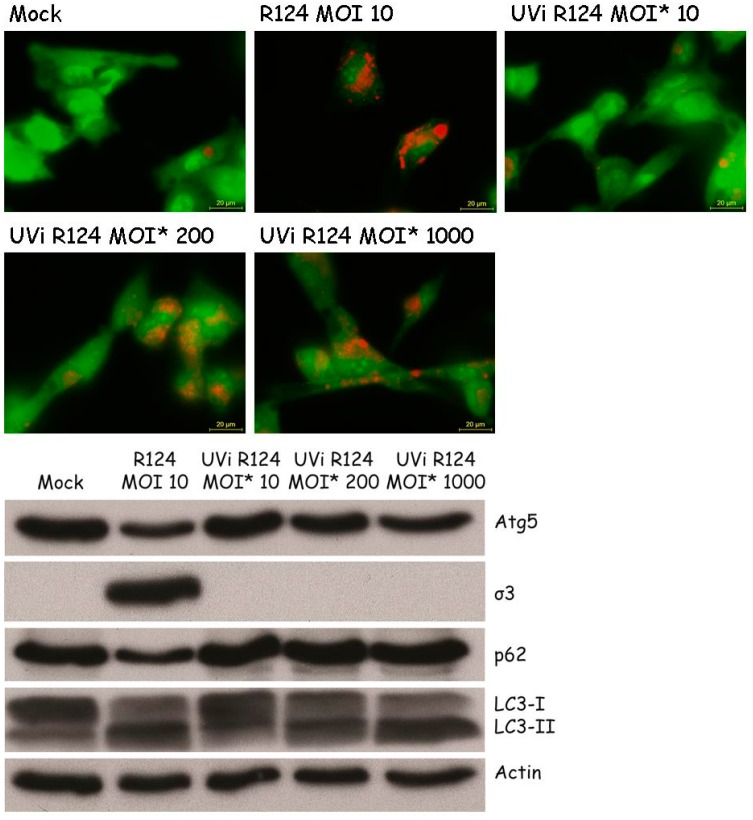
High amounts of UV-inactivated reovirus particles are needed to induce Atg5-Atg12 conjugation, LC3-I to LC3-II conversion, and acidic vesicular organelles in U87-MG cells. U87-MG cells were infected with R124 at an MOI of 10, or UV-inactivated R124 particles at an MOI* of 10, 200, or 1000. After 48 h, protein lysates were made in RIPA lysis buffer and analyzed for the expression of Atg5, σ3, p62, LC3, and Actin. Alternatively, cells were incubated with acridine orange and fluorescence microscopy images were taken.

Previous studies by Lv et al. and Arnoldi et al. on autophagy induction by Bluetongue Virus and Rotavirus showed that the induction of autophagy is dependent on virus replication [8,10]. To examine whether autophagy can be induced by replication-defective reovirus particles, U87-MG cells were exposed to UV-inactivated R124, or the corresponding replication-competent virus, at the indicated MOIs. The absence of infectious virus in the UV-inactivated R124 batch was confirmed by plaque assay on 911 cells (≤10 pfu/mL). The MOI of UV-inactivated reovirus particles is based on the infectious titer prior to UV inactivation, and is referred to as MOI*. Replication-competent R124 at an MOI of 10 clearly induced the appearance of enlarged acidic vesicles (Figure 8), indicative for autophagy. UV-inactivated virus particles gave a slight increase in the number of these autophagic vacuoles at an MOI* of 200 and 1000. Consistently, Atg5-Atg12 conjugation and LC3 lipidation were visible after infection with replication-competent R124 at an MOI of 10 or UV-inactivated R124 at an MOI* of 200 and 1000. This suggests that the reovirus genome is dispensable for the induction of autophagy. However, as high amounts of virus particles are needed for autophagy induction, productive R124 infection is more efficient in autophagy induction. Interestingly, levels of p62 were decreased only upon infection with replication-competent R124. Therefore, it seems that replication-incompetent R124 particles do not induce the later steps of the autophagic flux. Altogether, the obtained results demonstrate that a productive R124 infection is not strictly necessary, but R124 replication strongly stimulates the induction of autophagy.

## 4. Discussion

Oncolytic virotherapy using reovirus has entered a variety of clinical trials, which demonstrated both the safety and feasibility of the approach. Its efficacy has been limited in stand-alone therapies [1]. A better understanding of the reovirus-host interaction is crucial to improve therapeutic strategies. Here, we demonstrate that reovirus induces several key features of autophagy, evident by the appearance of double-membraned vesicles, an increase in acidic vesicles, LC3 conversion, p62 degradation, Atg5-Atg12 conjugation, and the formation of GFP-LC3 puncta in infected cells.

For other members of the Reoviridae family, it has been shown that autophagy enhances their replication. Bluetongue virus stimulates autophagy through the down-regulation of the autophagy-inhibiting Akt-TSC2-mTOR pathway and the up-regulation of the autophagy-stimulating AMPK-TSC2-mTOR pathway [25]. Autophagy was shown to enhance Bluetongue virus replication, as the pharmacological or siRNA-mediated inhibition of autophagy resulted in decreased virus replication, whereas inducing autophagy with Rapamycin (Rapa) led to an increase in viral yields [10]. Similarly, avian reovirus p17 non-structural protein has been shown to trigger autophagy through the activation of PTEN, AMPK, and PKR/eIF2α signaling pathways [9]. As in the study on Bluetongue virus, treatment with Rapa increased avian reovirus progeny, whereas inhibiting the autophagy pathway decreased virus replication. Studies on rotavirus have revealed that its viroporin NSP4 activates autophagy by stimulating the release of calcium from the ER into the cytoplasm, which leads to a CaMKK-β- and AMPK-dependent initiation of autophagy [26]. This process relies on the activity of PI3K, Atg3, and Atg5. As for Bluetongue virus and avian reovirus, inhibition of autophagy reduced the amount of infectious virus. Interestingly, a recently accepted manuscript showed contradictory findings that rotavirus activity can be reduced by blocking the PI3K-Akt-mTOR signaling pathway and thus stimulating autophagy [27]. Further research is warranted to gain more insight on this complex rotavirus-host interaction. For the interactions between mammalian reovirus and the autophagy machinery, not much is known. There are indications that mammalian reovirus induces autophagy in multiple myeloma cell lines, but the role of autophagy in mammalian reovirus infection remains unclear [11,12].

Our finding that GFP-LC3 puncta are formed in cells that show reovirus protein expression indicates that autophagy can be induced by the virus. For some viruses, it has been suggested that they use the autophagosome environment as a niche to replicate and/or be shielded from the cytoplasmic antiviral immunity [28,29,30]. Notably, Figure 1 shows that LC3 and the reovirus outer capsid protein µ1C do not overlap in our experiments, implying that the virus particles may exist in the vicinity of autophagosomes but not reside inside these vesicles. This finding is supported by the electron microscopy pictures, where no virions were detected inside autophagosomal structures.

The process of autophagy is characterized by the formation of autophagosomes, fusion of autophagosomes with lysosomes, and the degradation of the autolysosomal content [3]. LC3-II is localized to autophagosomes and therefore generally seen as a marker for autophagosome formation [21]. However, an increase in LC3-II can also be the consequence of a delay or a block in autolysosomal degradation. It is crucial to assess whether reovirus induces the full autophagic flux, including lysosomal degradation. For example, Influenza A virus blocks the fusion between autophagosomes and lysosomes with its matrix protein 2 [31]. The conventional method to study autophagic flux is through the use of BafA1. This compound blocks autophagosome-lysosome fusion or lysosomal acidification, and therefore should lead to a block in the degradation of autophagic proteins thus an increase in LC3-II and p62 [21]. It should be mentioned that BafA1 treatment can inhibit the entry steps of reovirus infection [32,33]. An effect of BafA1 on the reovirus-induced autophagy markers could thus potentially be attributed to an inhibition of viral entry. Therefore, we did not study the effect of adding BafA1 to reovirus-infected cells in our experiments. Nevertheless, it seems likely that reovirus induces the full autophagic flux, as we demonstrated that p62 is degraded upon reovirus infection.

In our experiments, we observed that not all of the autophagic machinery facilitates reovirus replication. Recent publications revealed that genes considered to have a specific role in autophagy can exert additional functions as well [34,35,36]. This underlines the importance of testing knock-out cell lines for multiple autophagy-related genes before drawing firm conclusions about whether autophagy is involved in the studied process. Our results indicate that the autophagic process is not strictly involved in the replication of reovirus, but that some functions of autophagy-related proteins are important for a productive virus infection. We show that a subset of autophagy-related proteins seems to facilitate reovirus replication: specifically, Atg3 and Atg5, which both are essential for the lipidation of LC3. This raises the question as to whether the formation of LC3-II rather than the process of autophagy facilitates the replication of reovirus. As LC3 lipidation does not strictly rely on the expression of Atg13, this would explain the contrasting result for that knock-out. Correspondingly, we noted that the cellular presence of LC3 puncta correlates with the presence of virus protein in the same cell, suggesting that they co-evolve. It remains to be determined whether the LC3 puncta consist of LC3-II or LC3-I. For Coronavirus, it has been demonstrated that virus infection in cell culture induces the formation of double-membraned vesicles coated with LC3-I [37]. However, we found that the levels of LC3-I decrease during reovirus infection, and the levels of LC3-II conversely increase. Therefore, it seems unlikely that LC3-I rather than LC3-II is detected as puncta.

Importantly, both Atg3 and Atg5 have been demonstrated to have additional functions besides facilitating autophagy [34,35,36]. It has been shown that Vaccinia Virus infection induces the formation of Atg3-Atg12, resulting in aberrant autophagosome formation [38]. This conjugate has previously been demonstrated to be involved in basal autophagic flux, mitophagy, and late endosome function [39,40,41]. It is widely known that reovirus entry relies on the maturation of endosomes, raising the possibility that in the absence of Atg3, entry of the virus is hindered [42,43]. Future studies are needed to investigate this possibility. Research on non-canonical roles of Atg5 has demonstrated that the Atg5-Atg12 conjugate can alternatively function to inhibit type I IFN production by directly interacting with RIG-I and IPS-1 [44]. Type I IFN is considered a general anti-viral response and is known to inhibit a productive reovirus infection [45]. Therefore, an Atg5-mediated decrease in type I IFN could explain the observed importance for reovirus replication.

Our findings that an MOI* of 10 of UV-inactivated reovirus fails to induce LC3 lipidation, Atg5-Atg12 conjugation, or an increase in acidic vesicular organelles demonstrate that a productive reovirus infection facilitates the induction of autophagy. It remains to be studied what specific step in the reovirus infection cycle is involved in the induction of autophagy. It has been shown that the apoptosis induction capacity of reovirus relies on σ1 attachment to cell surface molecules [46]. Contrastingly, for necroptosis induction by reovirus, both genomic RNA within incoming virus particles needs to be sensed as well as dsRNA must be formed [47]. Interestingly, for other members of the Reoviridae virus family, non-structural proteins were found to be associated with autophagy induction [27]. Our data indicate that this is not the case for reovirus. Though our results indicate that a productive reovirus infection facilitates the induction of autophagy, the replication process itself seems dispensable as we find that exposure of cells to a much higher MOI* (200 or 1000) of UV-inactivated virus is able to induce autophagic features. In this respect, it could be speculated that a specific reovirus capsid protein, or a set of proteins, is involved in the induction of autophagy.
